# Exploring the Core Functional Microbiota Related to Flavor Compounds in Douchi from the Sichuan–Chongqing Region

**DOI:** 10.3390/foods14050810

**Published:** 2025-02-26

**Authors:** Dawei Tu, Junhan Kang, Qingqing Li, Meilin Deng, Meiyan Liu, Wenjun Liu, Jian Ming, Margaret Brennan, Charles Brennan, Linfeng You

**Affiliations:** 1 School of Food Science and Engineering, Chongqing Technology and Business University, Chongqing 400067, China; 2017096@ctbu.edu.cn (D.T.); youlf@ctbu.edu.cn (L.Y.); 2 Chongqing Wanbiao Testing Technology Ltd., Chongqing 400714, China; kangjunhan@163.com (J.K.); 18723238669@163.com (Q.L.); deng0205076101@tom.com (M.D.); 13527569905@163.com (M.L.); 3 College of Food Science, Southwest University, Chongqing 400715, China; food_mj@swu.edu.cn; 4 Chongqing Academy of Chinese Materia Medica, Chongqing 400065, China; 5 School of Science, RMIT University, Melbourne, VIC 3000, Australia; margaretinpalmy@yahoo.co.nz (M.B.); charles.brennan@rmit.edu.au (C.B.)

**Keywords:** douchi, fermented soybean, amino acid, volatile component, microbial community, correlation

## Abstract

Douchi is a traditional Chinese fermented soybean product. In the Sichuan–Chongqing region, *Mucor*-type douchi was particularly famous for its distinctive flavor. Nevertheless, the association between microorganisms and douchi flavor is still poorly understood. In this study, high-throughput sequencing, amino acid analysis, and gas chromatography-mass spectrometry (GC-MS) were used to investigate the bacterial and fungal profiles as well as the flavor compounds (sixteen amino acids and one-hundred volatile flavor compounds) of seven different types of douchi. High levels of glutamic and aspartic acids were observed. Microbial analysis found that *Bacillus*, *Tetragenococcus*, *Weissella*, *Aspergillus*, *Mucor*, and *Penicillium* were the prime microorganisms. In total, 100 volatile components were detected; however, none of them was common to all the douchi products, although most volatile components had the aromas of flowers, fruits, caramel, and cocoa. An analysis of the flavor compounds was conducted using two-way orthogonal partial least-squares discriminant analysis (O2PLS-DA). Based on the analysis, it was found that Glu had negative correlations with most microorganisms, and *Aspergillus* had positive correlations with 2-pentylfuran and phenylacetaldehyde. This study provides a theoretical foundation for the regulation and enhancement of douchi flavor.

## 1. Introduction

Douchi is a traditional Chinese fermented soybean product with a pleasant flavor and is rich in nutrition. Depending on the fermentation’s main microorganism, it can be categorized into four different types [1,2,3], including *Mucor*-type douchi, *Aspergillus*-type douchi, bacterial-type douchi, and *Rhizopus*-type douchi. In China, *Mucor*-type douchi is predominantly produced in southwest areas, with typical products being Yongchuan douchi and Tongchuan douchi.

Traditional douchi has been made by steeping, stewing, cooling, zhiqu, stirring salt and post-fermentation and is produced via traditional natural fermentation, with most of the microorganisms derived from the natural environment and raw materials, and its post-fermentation time takes more than 10 months. In addition, because *Mucor* grows only at low temperatures, traditional douchi can be produced only from November through February [1,4,5]. As a result, traditional natural zhiqu douchi was unable to satisfy the market’s needs; thus, the industrialized *Mucor*- and *Aspergillus*-type douchis could develop.

Aroma determination has become a hot research topic. Headspace solid-phase micro extraction (HS-SPME), stir bar sorptive extraction (SBME), and supercritical fluid extraction (SFE) are mainly extraction methods for sample pretreatment [6,7]. The advantages of SPME are its simplicity, ease of automation, and robustness, and it is used for determining the flavors of volatile and semi-volatile compounds [6]. Perestrelo [8] et al. chose six types of commercially available silica SPME fibers and found that divinylbenzene/carboxen/polydimethylsiloxane (DVB/CAR/PDMS) fiber had the highest extraction efficiency for the analytes released into the headspace. Also, other researchers used DVB/CAR/PDMS to evaluate the flavor quality [9,10].

Researchers have been investigating the roles of different starter cultures in a range of fermented foods and how they can affect the sensory quality of fish sauces [11] and Chinese fermented soybeans, such as douchi and sufu [12,13], and how the components of these foods can affect the human microbiota. Most recent research has focused on the roles of microorganisms in the quality of fermented products. For instance, Li [14] conducted a study on the association between microorganisms and flavor formation in *Aspergillus*-type douchi through 16S rRNA sequencing and SPME-GC-MS and found that bacteria were the dominant microorganism. The researchers evaluated the key model of bacteria and flavor components by O2PLS analysis and found that the main volatile flavor components were aldehydes, ketones, and acids for *Staphylococcus*, ketones and pyrazines for *Bacillus*, and acids and aldehydes for *Lactobacillus*. A study by Li [15] explored the relationship between the top 10 microorganisms in bacteria and fungi and flavor components during the natural fermentation of red sufu from Chaling and found that *Leuconostoc*, *Lactobacillus*, *Weissella*, *Debaryomyces*, *Tausonia*, and *Pichia* were the main microorganisms responsible for the flavor formation of sufu. Zhang [1] compared the quality and microbial communities between natural-type and artificial-type douchis and found that the microflora of the natural-type douchi were more abundant and diverse. Wang [13] et al. investigated the effect of the microbial community on the volatile aromatic substances of *Mucor racemosus* during sufu fermentation. They found that *Lactobacillus*, *Pseudomonas*, and so on primarily influenced the synthesis of acids and alcohols.

In this study, seven different types of douchi were used as raw materials from Sichuan–Chongqing. High-throughput sequencing, flavor chemistry, and correlation analysis were used to establish the correlation between the microbiota and flavor substances in douchi and to screen out potential flavor-related microorganisms. These will provide a theoretical research basis for the later screening of flavor-producing bacteria and validation in rapid fermentation models, as well as a better understanding of the relationship between the microbiota and flavors.

## 2. Materials and Methods

### 2.1. Preparation and Collection of Douchi

All the douchi samples were collected from different manufacturers in Sichuan Province and Chongqing. All the samples were grouped separately from DC-1 to DC-7, and the distribution is shown in Table 1. After the samples were collected, they were transported at −80 °C for DNA extraction and at 4 °C for physicochemical analysis after homogenization.

### 2.2. Determination of the Microbial Diversity in the Samples

Microbial DNA was extracted from all the samples using a FastDNA Spin Kit (MP Biomedicals, Santa Ana, CA, USA). A Nanodrop 2000 spectrophotometer (Thermo Scientific, Wilmington, DE, USA) was used to measure the final DNA concentration and purity. The DNA quality was checked using 0.8% agarose gel electrophoresis.

The 16S primers were 338F (5′-ACTCCTACGGGAGGCAGCA-3′) and 806R (5′-GGACTACHVGGGTWTCTAAT-3′). The ITS primers were ITS1-F-KYO2 (TAGAGGAAGTAAAAGTCGTAA) and ITS86R (TTCAAAGATTCGATGATTCAC). PCR amplification was performed under the following conditions: initial denaturation at 95 °C for 3 min, followed by 35 cycles of denaturation at 95 °C for 30 s, annealing at 54 °C for 30 s, and extension at 72 °C for 45 s, with a final elongation at 72 °C for 10 min. The PCR products were extracted by 2% agarose gel and further purified using the AxyPrep DNA Gel Extraction Kit (Axygen Biosciences, Union City, CA, USA).

The amplicon library was generated using a TruSeq Nano DNA LT Library Prep Kit (Illumina, San Diego, CA, USA). High through OTU sequencing was performed using an Illumina HiSeq platform (Sichuan PANOMIX Biotech Co., Ltd., Chengdu, China).

Low-quality sequences were removed by QIIME2 (Vision 2019.4) (https://view.qiime2.org/, accessed on 23 December 2024). The raw 16S RNA and ITS gene sequences were denoised or clustered into amplicon sequence variants (ASVs) using QIIME2 (Vision 2019.4). The alpha diversity, such as the Chao 1 richness and Shannon diversity indices, was evaluated using QIIME2.

### 2.3. Determination of Amino Acid Levels

Samples (100 mg) were hydrolyzed by adding 15 mL of 6 M HCl solution at 110 ± 1 °C for 22 h. Then, 1 mL of the hydrolysate was concentrated, a sodium citrate buffer solution was added, and the mixture was passed through a 0.22 μm filter membrane. An L-8900 amino acid analyzer (Hitachi, Tokyo, Japan) was operated according to the manufacturer’s instructions.

### 2.4. Volatile Compound Analysis

The volatile compounds of the douchi were tested using a Shimadzu QP2020 NX gas chromatography–mass spectrometer (GC-MS, Shimadzu, Kyoto, Japan). Sample pretreatment was conducted based on the method described by Li [16], with minor modifications. Douchi samples (3 g) were put into 30 mL headspace vials, an internal standard (1,3-dichlorobenzene, 100 μg/mL, 100 μL) was added, and the headspace vials were immediately sealed. Solid-phase micro-extraction (SPME) was performed at equilibrium at 60 °C for 20 min, adsorption was performed at 60 °C for 30 min using a 50/30 μm DVB/CAR/PDM fiber (Supelco, St. Louis, MO, USA), manual injection and desorption were performed at 250 °C for 5 min.

Chromatographic conditions: The volatile compounds were separated by an SH-Rxi-5Sil MS column (30 m × 0.25 mm, 0.25 μm; Shimadzu, Kyoto, Japan). Helium was used as a carrier gas with an average linear velocity of 1.0 mL/min. The injector temperature was 230 °C. The column temperature was initially 40 °C and was held for 3 min. Then, the temperature was increased to 105 °C at a rate of 4 °C/min. Then, the temperature was increased to 150 °C at a rate of 5 °C/min. Finally, the temperature was increased to 240 °C at 10 °C/min and maintained there for 15 min.

Mass spectrometry conditions: The electron ionization (EI) was set at 70 eV. The ion-source temperature was 250 °C. The scanning mode was the full-scan mode (SCAN), with a mass-scanning range that was configured from m/z 35 to 500.

The volatile compounds were preliminarily identified by comparing their mass spectra with the mass spectral library (NIST 2017), which was at least 80%, and matching the retention indices (RIs), retention times, and mass spectra, which were calculated for n-alkanes (C7-C40, Shanghai, China). Each experiment was repeated three times.

The components were quantified according to the semi-quantitative method. The formula for calculating the concentration of the flavor compounds is as follows:

(1) $$W_{i}=\frac{A_{i}\times W_{0}}{A_{0}}$$

where W*_i_* is the sample’s mass concentration, *A_i_* is the sample’s peak area, *W*_0_ is the standard mass concentration, and *A*_0_ is the standard peak area.

The odor activity value (OAV) was determined by dividing the concentration of a volatile compound by its perception threshold.

### 2.5. Statistical Analysis

The data were measured in triplicate and expressed as mean values with standard deviations. Origin 2021 software (OriginLab, Northampton, MA, USA) was used for data visualization and basic statistical analyses. O2PLS-DA was conducted to analyze the volatile compounds’ data using SIMCA 14 (demo v.1.0.1) (Umetrics AB, Umea, Sweden).

## 3. Results and Discussion

### 3.1. Alpha Diversity in Different Fermented Douchi Products

The alpha diversity index reflects the richness and evenness of the microbial communities for each group, including the Shannon, Simpson, and Chao indices [15,17,18]. As shown in Table 2, the goods coverage of the bacteria and fungi in the douchi samples was above 0.9999, which indicated that the majority of the microorganisms were detected. The diversity (Shannon and Simpson indices) and richness (Chao index) of the microbial communities in the samples were determined using standardized sequences [19,20]. The Shannon and Simpson indices indicated that the diversity of DC-5 was the highest among the bacteria-fermented material, while DC-2 was the highest in the fungi-fermented products.

### 3.2. Microbial Community Composition in Douchi Products

**Figure 1:**
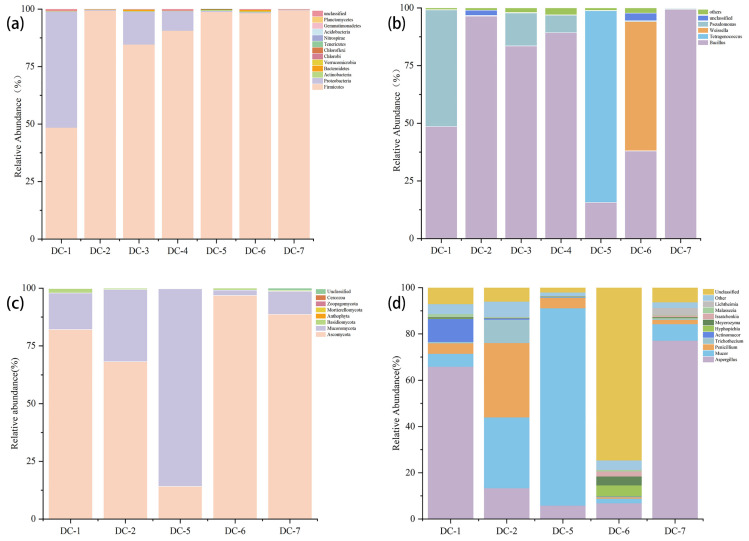
The relative abundance changes of the bacteria in douchi samples at phylum (**a**) and genus levels (**b**) and fungi in douchi samples at phylum (**c**) and genus levels (**d**).

The microbial community composition was different among the douchi products. As shown in Figure 1a, *Firmicutes* was the dominant microorganism, followed by *Proteobacteria*. It is worth noting that *Firmicutes* made up nearly 99% of the community in DC-2, DC-5, DC-6, and DC-7. The ratio of *Firmicutes* to *Proteobacteria* was almost 1:1 in DC-1, while it was 6:1 in DC-3 and 10:1 in DC-4. The fungi in DC-3 and DC-4 were not amplified because there were fewer of them in the sample. At the phylum level (Figure 1c), there were three phyla with relative abundances of >1%, including *Ascomycota*, *Mucoromycota*, and *Basidiomycota*. These phyla were also found to be major components of microbial flora in Chinese fermented vegetable foods, fermented fish, and chestnut rice wine [19,21,22].

At the genus level of bacteria (Figure 1b), the dominant genus was *Bacillus*, accounting for 15.68–99.34% in all the samples. The genera with relative abundances of >1% were *Bacillus*, *Pseudomonas*, *Tetragenococcus*, and *Weissella*, while the rest of the genera had very low abundances. *Bacillus* spp. were also the dominant bacterial taxa in many fermented soybean products and the wine industry [21], and they contributed flavor through amylase and protease activities. Tan [23] reported that *Bacillus* may prefer anaerobic or microaerophilic conditions. In our study, the abundances of *Bacillus* in DC-2, DC-3, DC-4, and DC-7 exceeded 80%; it may be related to douchi that was not agitated during the post-fermentation period. Research has also indicated that *Pseudomonas* improves the flavor of douchi by producing more kinds of protease, lipase, and lecithin enzymes [24], and in our study, the relative abundances of *Pseudomonas* in DC-1 (50.54%), DC-3 (14.27%), and DC-4 (7.54%) were all above 1%. *Tetragenococcus* and *Weissella* were lactic acid bacteria (LABs) that have been detected in a variety of fermented foods and play important roles in flavor generation, and these were involved in the production of lactate, acetate, and ethanol [23]. *Tetragenococcus* is a halophilic LAB, which has been noted as being the dominant bacteria of traditional fermented soybean products in China, Japan, South Korea, and other southeast Asian countries. In our study, DC-5 had a high level of *Tetragenococcus* (83.08%); meanwhile, *Weissella* (56.04%) was high in DC-6. Some bacterial species were unique in each sample because of different raw materials, processing technologies, and fermentation environments [25].

The fungal phylum level is shown in Figure 1d; the number of major fungi was 10, involving *Aspergillus*, *Mucor*, *Penicillium*, and *Trichothecium*. Compared with the bacterial variation, the variation in the fungal composition was more complex, with the most dominant microorganisms varying from sample to sample. In DC-1 and DC-7, *Aspergillus* was the dominant fungus, accounting for 65.88% and 77.18%. *Aspergillus* has been shown to be a major fungal group in ganjiang (a Korean traditional soy sauce) [26], and it secreted a variety of enzymes that contribute to the texture and flavor of fermented foods [27]. Kim [28] claimed *Aspergillus* and *Zygosaccharomyces* were the predominant fungi in Chinese fermented soybean paste, which was somewhat different from this study. Meanwhile, it has been reported that *Mucor* plays a vital role in fermented soybean products in generating a huge number of proteases to degrade proteins [29]. In DC-2, *Mucor* (30.63%) and *Penicillium* (32.63%) were the prime fungi, while *Mucor* presented the highest relative abundance (85.33%) in DC-5. In DC-6, there were lots of fungi that were not classified at the class level.

According to these findings, we found that the structure of the bacterial flora in these douchi was highly similar, while the fungal structure was different, which indicated different processes had less effect on their bacterial structure and more effect on their fungal structure.

### 3.3. Bacterial and Fungal Interaction Analysis

**Figure 2:**
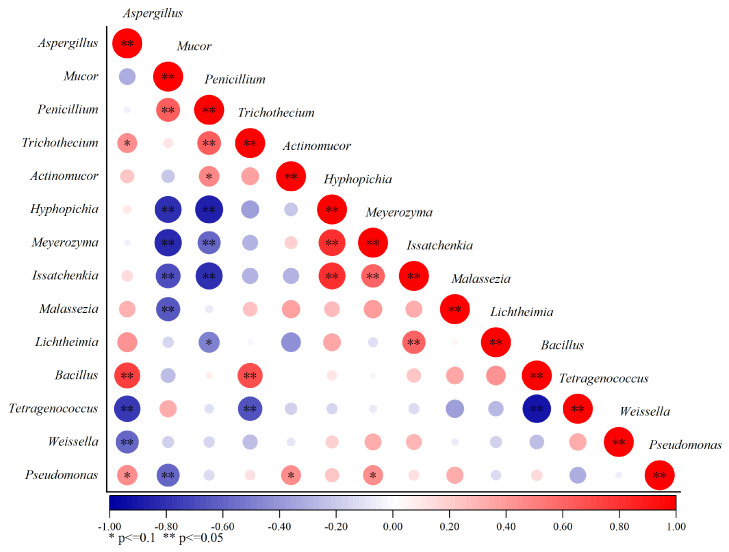
Correlation analysis among microorganisms. The color indicates the correlations between microorganisms, with higher values from negative (blue) to positive (red) and darker colors indicating a stronger correlation. Significant values were shown as * *p* < 0.1; ** *p* < 0.05.

Microbial interaction is considered to be an important factor affecting fermented soy beans’ microbial structure [30,31]. Positive and negative correlations were used to indicate symbiotic and antagonistic relationships through Spearman’s rank correlation coefficient (Figure 2). *Aspergillus* exhibited positive correlations with fungal genera, except for *Mucor*, which was contrary to previous research findings [32]. *Tetragenococcus* exhibited negative correlations with most microbials, particularly strong exclusion with *Aspergillus*, *Trichothecium*, and *Bacillus*. *Bacillus* exhibited co-occurrence relationships with most fungal bacteria and exclusive relationships with *Weissella* and *Tetragenococcus*.

### 3.4. Amino Acid Analysis

**Figure 3:**
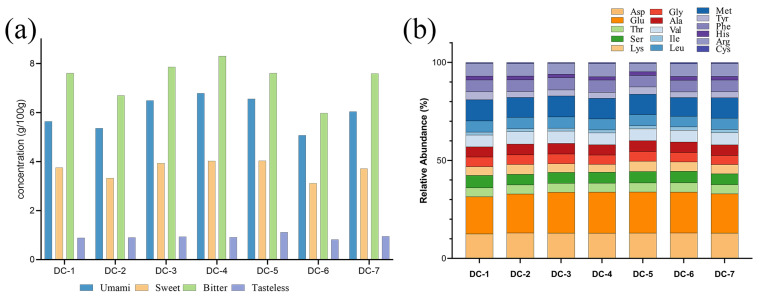
The contents of amino acids in douchi samples. (**a**) The amino acids of different flavors; (**b**) the relative abundances of amino acids.

According to previous studies, free amino acids provide flavor and color to foods by participating in complex reactions, such as Maillard reactions [33,34]. Free amino acids in different douchis are shown in Figure 3. A total of sixteen free amino acids were detected and classified into four categories: umami amino acids (Asp and Glu), sweet amino acids (Thr, Ser, Gly, and Ala), bitter amino acids (Val, Ile, Leu, Met, Tyr, Phe, His, and Arg), and tasteless (Lys and Cys) [35]. The total contents of each douchi ranged from 14.99 to 20.03 g/100 g (Table S1); among them, bitter-amino-acid contents ranged from 5.98 to 8.30 g/100 g, while umami amino acids were 5.08–6.79 g/100 g (Figure 3a). As shown in Figure 3a, DC-4 had the most umami, sweet, and bitter amino acids, whereas DC-6 had the least. Figure 3b illustrates the relative abundances of amino acids, which were very similar in each douchi sample. Among them, Glu accounted for the highest proportion, reaching 20%, followed by Asp and Met, accounting for 13% and 10%, respectively. This finding is similar to previous research showing that soy proteins with plentiful Gul and Asp, such as in soybean fermentation products, like douchi, soy sauce, and dajiang, usually exhibit an excellent umami taste.

The taste activity value (TAV) was used to evaluate the contributions of taste-active compounds, and a TAV of ≥1 was regarded as significant for the overall taste impact. In this study, Glu’s TAV was the highest, as well as those of most umami and bitter amino acids. Zhou [2] found that umami amino acids (Glu and Asp) were one of the significant contributors to the taste of soybean fermentation products. A higher proportion of umami amino acids contributed to the higher umami score of the douchi. Nevertheless, excesses of bitter amino acids might have a negative influence on the umami-amino-acid taste stimulation, affecting its umami score. Another study claimed that high salt and acidity levels inhibited the perception of bitterness, despite the presence of bitter-causing amino acids [20].

### 3.5. Volatile Components

**Figure 4:**
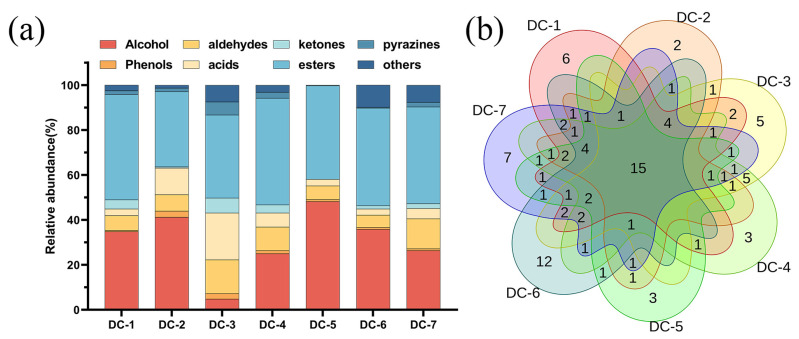
The composition of the volatile components in the douchi samples. (**a**) The relative abundances of volatile components; (**b**) Venn analysis.

**Figure 5:**
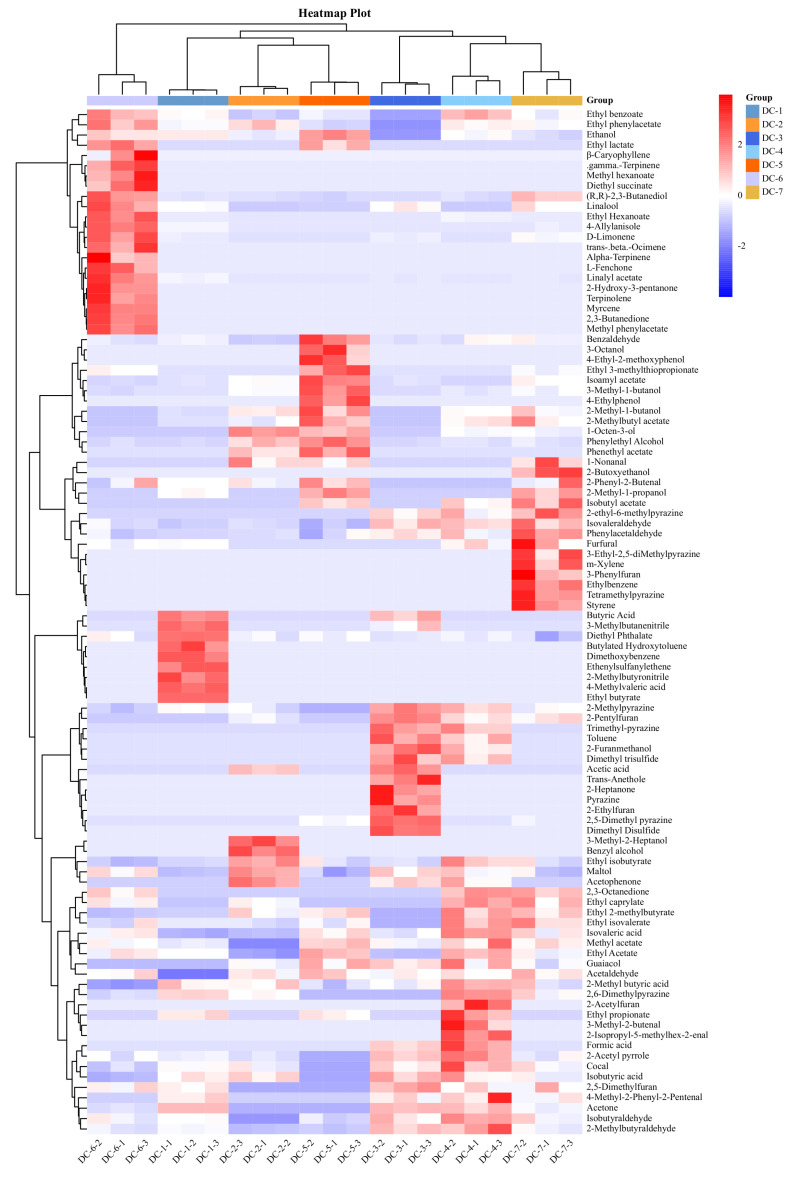
Heatmap of the volatile substances in different douchis.

Volatile flavor substances are crucial for flavor formation in fermented soybean products and are significantly influenced by microbial diversity, as well as their metabolic effect during the fermentation process. The contents of volatile compounds in the douchi samples are shown in Figure 4. A total of one-hundred volatile compounds were detected, including twelve alcohols, five phenols, thirteen aldehydes, seven acids, seven ketones, twenty-two esters, eight pyrazines, and twenty-six others (Table S2). The proportions of various volatile compounds varied when the content of each volatile component was normalized. As is shown in Figure 4a, esters and alcohols were predominant. Furthermore, Figure 4b depicts the Venn diagram of the number of volatile compounds in each douchi. Fifteen volatile compounds were common among all seven samples, and six, two, five, three, three, twelve, and seven volatile compounds were unique from DC-1 to DC-7. In addition, DC-7 (57) had the highest number of volatile compounds, followed by DC-6 (52) and DC-4 (50). DC-2 (39) and DC-5 (39) had the lowest numbers of volatile compounds (Figure 5).

From the heatmap visualization of the flavor substances in the douchis (Figure 5), DC-2, DC-5, and DC-1 were grouped together, while DC-4, DC-7, and DC-3 were grouped together, and DC-6 was classified in a separate category. Ethyl benzoate, ethyl phenylacetate, ethyl lactate, and ethyl hexanoate had the highest contents in DC-6. Furthermore, some olefin compounds, like myrcene, alpha-terpinene, and gamma-terpinene, were detected only in DC-6. 1-Octen-3-ol, phenylethyl alcohol, and phenethyl acetate, were high in DC-2 and DC-5, while pyrazine compounds were found to be more abundant in DC-3 and DC-4.

Esters were one of the main compounds in the douchis, which is homologous to the results in the previous coverage of fermented products [33,36]. A total of 22 esters were detected (Table S2). They were mainly produced by the esterification of free fatty acids with organic acids and alcohols in douchi as precursors [37]. Among the esters, the contents of ethyl acetate, ethyl benzoate, ethyl phenylacetate, and diethyl phthalate were the most dominant, being formed in the post-fermentation process, bringing sweet, floral, fruity, and other good flavors [38].

Alcohols were one of the main compounds in the douchis, forming during fermentation, and their synthesis is linked to the amino-acid metabolism and sugar metabolism of yeast [39]. Ethanol, 1-octen-3-ol, phenylethyl alcohol, and 3-methyl-1-butanol were observed to be the highest-abundance alcohol components, and these are often described as having mushroom-like, fruity, and honey-like flavors [40]. Ethanol was the dominant alcohol in all the douchi products; it may be relevant to adding baijiu during the mixing and the metabolism of microorganisms [24]. 1-Octen-3-ol was found to be a common flavor compound in fermented soybean products in previous studies [41], but in this study, it was only found in DC-2 and DC-5. 2,3-Butanediol is one the characteristic flavor substances in fermented products and displays a sweety fruity aroma. These contributed greatly to the formation of the douchi flavor.

In this study, eight pyrazines have been detected, such as 2-methylpyrazine, 2,6-dimethylpyrazine, 2,5-dimethyl pyrazine, 2-ethyl-6-methylpyrazine, trimethyl-pyrazine, pyrazine, 3-ethyl-2,5-dimethylpyrazine, and tetramethylpyrazine. Pyrazines are released from the non-enzymatic browning in cooked soybeans or microbial metabolism, which occurs during the degradation of protein into amino acids when the fermentation time begins [3,38,42]. The presence of 2-methylbutyraldehyde, benzaldehyde, and phenylacetaldehyde contributed to the aromas of nuts, almonds, and flowers.

The OAV was used to detect the contributions of the volatile compounds to the overall aroma profile [43]. The greater the OAV value, the greater the contribution to the overall flavor. In our study, twenty-six kinds of flavor compounds were identified as the key flavor compounds, with OAVs of >1 in the above four samples, including nine esters, six alcohols, six aldehydes, three acids, one phenol, and one furan (Table 3). The OAV values of the volatile compounds, such as isobutyraldehyde, 2-methylbutyraldehyde, isovaleric acid, ethyl benzoate, and ethyl phenylacetate, in DC-4 were higher than those of the volatile compounds in the other samples, indicating the rich aroma of DC-4. Isovaleraldehyde, 2-methylbutyraldehyde, phenylacetaldehyde, isovaleric acid, and ethyl isobutyrate were present in all the samples with an OAV value of >100; this gave the douchis fruity, caramel-like, floral, and pungent aromas. Isovaleraldehyde, 2-methylbutyraldehyde, phenylacetaldehyde, isovaleric acid, ethyl isobutyrate 1-octen-3-ol, ethyl benzoate, and ethyl isovalerate were present as flavor compounds in the douchis. Phenylacetaldehyde has been identified as the critical aromatic compound responsible for the “honey-like” aroma in sufu, douchi, soy sauce, and other fermented soybean products and was formed in the Ehrlich pathway [41]. Benzaldehyde and benzeneacetaldehyde have been recognized as key flavor contributors in douchis [44] and soy bean pastes [45]. 1-Octen-3-ol is a common flavor compound in fermented soybean products, especially in douchis, and is one of the main constituents during koji making [3]. In this study, 1-octen-3-ol was detected in DC-2, DC-4, DC-6, and DC-7.

### 3.6. Multivariate Statistical Analysis of Volatile Components and Amino Acids

**Figure 6:**
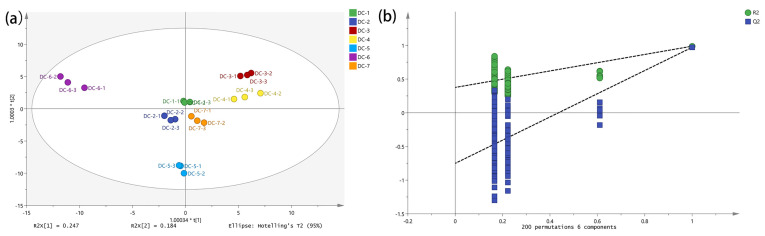
O2PLS-DA results for different douchi products. (**a**) The O2PLS-DA scores’ plots of the volatile compounds and amino acids in the douchi products; (**b**) model cross-validation results.

Figure 6 illustrates the model cross-validation results of the flavor substances after 200 substitution tests. In model of the flavor substances, R^2^X = 0.862, R^2^Y = 0.984, and Q^2^ = 0.957, which were all >0.5 and indicated that the model was stable and reliable. The intersection of the regression line of Q^2^ and the *Y*-axis was on the negative half-axis, indicating that the model was effective without overfitting. It could be used to reflect the flavor substance. The O2PLS-DA boxplots of the flavor substances in the samples are shown in Figure 6a. The differences among the various products could be effectively analyzed using O2PLS-DA. DC-3 and DC-4 were positioned in the first quadrant, DC-6 resided in the second quadrant, DC-2 and DC-5 were located in the third quadrant, and DC-7 is found in the fourth quadrant. Although DC-1, DC-2, DC-4, and DC-7 were not in the same quadrant, they are related.

Through O2PLS-DA, a variable-importance-in-projection (VIP) value could be obtained for each metabolite. The higher the VIP value, the greater the contribution of the substance to distinguish the groups. Thus, we usually took the VIP value as one of the important indicators when selecting different metabolites [47,48]. We found that the VIP values of twenty-seven bacterial genera and fifty-six volatile compounds were greater than one.

We selected the flavor substances which had both VIP > 1 and OAV/TAV > 1 to establish the correlation analysis with microorganisms, including one amino acid (Glu) and twenty-two volatile components (ethanol, 3-methyl-1-butanol, (R,R)-2,3-butanediol, linalool, phenylethyl alcohol, 1-octen-3-ol, isobutyraldehyde, isoamyl acetate, benzaldehyde, phenylacetaldehyde, acetaldehyde, isovaleric acid, ethyl acetate, ethyl isobutyrate, ethyl isovalerate, isoamyl acetate, ethyl phenylacetate, ethyl benzoate, diethyl phthalate, ethyl caprylate, and 2-pentylfuran).

### 3.7. Correlation Analysis Between Flavor Compounds and Microorganisms

**Figure 7:**
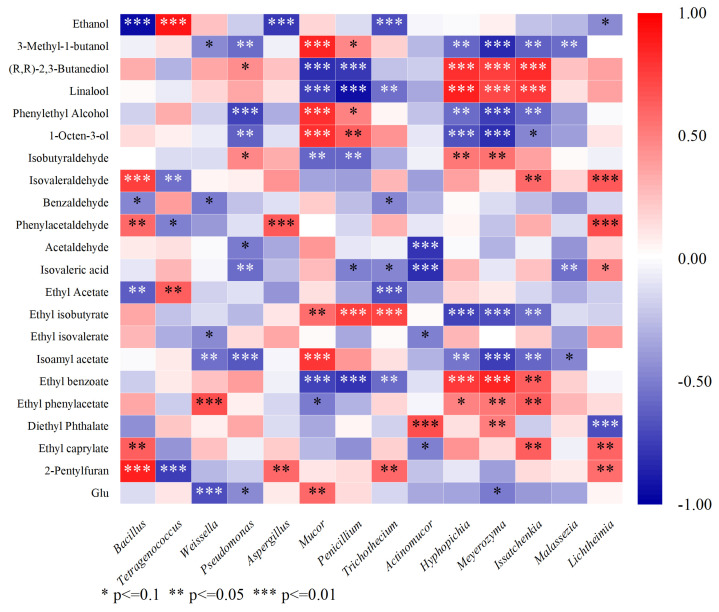
Spearman’s correlation heatmap between the dominant microbial community and volatile metabolites of the douchi products. The correlation coefficients appear in different colors; the right side of the legend is the color range of the different values. Significant values were shown as * *p* < 0.1; ** *p* < 0.02, *** *p* < 0.01.

Spearman’s correlation analysis was used to investigate the correlations between the volatile components and microorganisms in douchis [49]. In Figure 7, a red hue suggests a positive correlation; the darker the color indicates the stronger the positive correlation (a correlation coefficient closer to +1). A blue hue suggests a negative correlation, and the darker the color indicates the stronger the negative correlation (a correlation coefficient closer to −1).

Among the bacteria, *Bacillus* showed the highest number of highly positive correlations (*r* > 0.7) to flavor compounds (4), followed by *Tetragenococcus* (2), *Pseudomonas* (2), and *Weissella* (1), while *Pseudomonas* was highly negatively correlated with 7 flavor compounds. Furthermore, *Bacillus* was significantly positively correlated with isovaleraldehyde, phenylacetaldehyde, ethyl caprylate, and 2-pentylfuran, which indicated its important contribution to the metabolism of the volatile compounds in the douchi. Previous studies had shown that *Tetragenococcus* strongly influences the flavors of fermented foods [50,51]. In this study, *Tetragenococcus* showed significantly positive correlations with ethanol and ethyl acetate. On the negative side of the correlation, *Pseudomonas* was the more prominent bacterial genera, which is a finding similar to those in previous research [49]. Of these, *Pseudomonas* was strongly negatively correlated with 1-octen-3-ol, isovaleric acid, isoamyl acetate, acetaldehyde, and phenylethyl alcohol.

In fungi, *Mucor*, *Meyerozyma*, and *Issatchenkia* showed the highest number of highly positive correlations (*r* > 0.7) to flavor compounds (6), followed by *Hyphopichia* (5), *Penicillium* (4), and *Aspergillus* (2). *Mucor* and *Penicillium* were strongly positively correlated with 1-octen-3-ol and ethyl isobutyrate. Meanwhile, *Hyphopichia*, *Meyerozyma*, and *Issatchenkia* were strongly positively correlated with ethyl benzoate and isobutyraldehyde and negatively with ethyl isobutyrate and 1-octen-3-ol. It is interesting that Glu only had a positive correlation with *Mucor*.

Previously, studies on flavor-producing bacteria in douchis have focused almost exclusively on *Staphylococcus*, *Escherichia–Shigella*, and *Puccinia* [4]. Studies on flavor-producing fungi in douchis have shown that *Aspergillus* and *Penicillium* were positively correlated with citric acid, ethyl acetate, phenol, and benzaldehyde, and *Debaryomyces* was positively associated with ethyl butyrate and Glu [4,38], whereas in this study, many other bacteria and fungi were found to correlate strongly with volatile compounds. It is an interesting finding, and some applications deserve to be carried out to verify whether these bacteria do have the ability to improve the flavor of douchis.

## 4. Conclusions

This study evaluated the correlations between the microbiota and flavor compounds of seven different douchis. Among them, *Bacillus*, *Pseudomonas*, *Tetragenococcus*, and *Weissella* were the predominant bacteria, while *Aspergillus*, *Mucor*, *Penicillium*, and *Trichothecium* were the major fungi. As for amino acids, the content of Glu was the most prevalent, followed by Asp and Cys. Bitter-causing amino acids had the highest concentrations, which was consistent with the result after sensory tasting. As for the volatile components, 100 volatile compounds were spotted in total. Despite having few volatile components in common, all the douchi appear to have fruity, caramel, floral, and cocoa aromas.

The research was successful in developing a novel correlation model to visualize the complex relationship among the amino acids, volatile components, and microorganisms. Glu had negative correlations with lots of microorganisms. 3-Methyl-1-butanol, phenylethyl alcohol, 1-octen-3-ol, ethyl isobutyrate, and isoamyl acetate had positive correlations with *Mucor* and *Penicillium* and were negatively correlated with *Hyphopichia*, *Meyerozyma*, and *Issatchenkia*.

The present study will establish a solid foundation for the further validation of correlations between microorganisms and flavor while also enhancing the microbial spectrum information pertaining to douchis from different corporations. These findings serve as a fundamental basis for selecting the optimal flavor-producing strain for douchi production.

## Figures and Tables

**Table 1:** The locations of the seven samples.

Sample	Location
DC-1	Chongqing
DC-2	Chongqing
DC-3	Chongqing
DC-4	Sichuan
DC-5	Chongqing
DC-6	Sichuan
DC-7	Chongqing

**Table 2:** Alpha diversity indices of the different fermented douchi products.

Type	Sample	Chao1	Simpson	Shannon	Ace	Goods_Coverage
bacteria	DC-1	129.96	0.69	2.11	117.83	0.9999
	DC-2	141.36	0.44	1.70	135.80	0.9999
	DC-3	256.19	0.53	1.79	234.57	0.9999
	DC-4	106.60	0.54	2.06	99.57	0.9999
	DC-5	143.56	0.77	2.70	135.87	0.9999
	DC-6	200.98	0.53	1.85	189.83	0.9999
	DC-7	112.40	0.59	2.56	105.93	0.9999
fungi	DC-1	120.31	2.13	0.55	121.56	0.9999
	DC-2	113.65	3.13	0.81	114.21	0.9999
	DC-5	97.78	0.97	0.26	95.72	0.9999
	DC-6	105.63	1.93	0.51	98.23	0.9999
	DC-7	92.88	1.61	0.40	94.80	0.9999

**Table 3:** The contents of the major volatile components in the douchi products (g/100 g).

Compound	R.I. calc. ^a^	R.I. Ref. ^b^	Odor Description ^c^	Threshold ^d^ mg/kg	DC-1		DC-2		DC-3		DC-4		DC-5		DC-6		DC-7	
					Content (mg/kg)	OAV	Content (mg/kg)	OAV	Content (mg/kg)	OAV	Content (mg/kg)	OAV	Content (mg/kg)	OAV	Content (mg/kg)	OAV	Content (mg/kg)	OAV
Ethanol	/	ND	Wine, Pungent Flavor	0.62	11.91 ± 0.02	19.21	8.03 ± 0.41	12.95	0.56 ± 0.08	0.9	9.92 ± 0.84	16.01	19.42 ± 1.57	31.32	13.33 ± 1.64	21.5	6.36 ± 0.91	10.25
3-Methyl-1-butanol	731	734	Alcohol, Fruity, Banana	0.0061	0.08 ± 0.01	13.21	0.32 ± 0.05	52.33	0.04 ± 0.01	7.23	0.14 ± 0.04	22.23	1.59 ± 0.31	259.94	0.04 ± 0.03	7.21	0.29 ± 0.1	47.43
2-Methyl-1-butanol	735	736	Fish Oil, Green, Malt, Onion, Wine	0.14	0.05 ± 0.01	0.39	0.19 ± 0.03	1.37	ND	ND	0.14 ± 0.01	0.97	0.38 ± 0.16	2.74	ND	ND	0.17 ± 0.1	1.22
Linalool	1101	1104	Coriander, Floral, Lavender, Lemon, Rose	0.0024	0.17 ± 0.01	71.29	ND	ND	0.25 ± 0.05	102.8	ND	ND	0.01 ± 0.01	5.84	0.68 ± 0.2	284.78	0.26 ± 0.09	109.09
Phenylethyl Alcohol	1114	1114	Fruit, Honey, Lilac, Rose, Wine	0.012	0.07 ± 0.01	6.05	0.72 ± 0.16	59.91	0.06 ± 0.03	5.38	0.13 ± 0.03	10.83	1.15 ± 0.16	95.9	0.09 ± 0.04	7.12	0.15 ± 0.06	12.1
1-Octen-3-ol	982	986	Mushroom-like	0.001	ND	ND	1.13 ± 0.08	1129.17	ND -	ND	0.31 ± 0.04	309.04	0.85 ± 0.11	852.71	ND	ND	0.27 ± 0.08	270.4
Guaiacol	1086	1096	Smoky, Burning	0.0095	ND	ND	0.09 ± 0.02	9.62	0.15 ± 0.03	16.07	0.2 ± 0.11	20.64	0.18 ± 0.08	18.6	ND	ND	0.08 ± 0.04	8.08
Isobutyraldehyde	/	552	Burnt, Caramel, Cocoa, Green, Malt	0.001	0.18 ± 0.01	178.75	ND	ND	0.24 ± 0.07	237.35	0.34 ± 0.03	344.32	0.11 ± 0.03	105.22	0.16 ± 0.05	159.63	0.18 ± 0.07	183.12
Acetaldehyde	/	/	Floral, Green Apple	0.0027	ND	ND	0.22 ± 0.04	81	0.2 ± 0.03	75.08	0.21 ± 0.03	78.69	0.26 ± 0.06	97.15	0.22 ± 0.04	82.45	0.26 ± 0.06	95.87
Isovaleraldehyde	/	649	Fruity, Chocolate	0.002	0.27 ± 0.02	135	0.32 ± 0.02	160	0.68 ± 0.12	340	0.64 ± 0.06	320	0.21 ± 0.07	105	0.35 ± 0.07	175	0.78 ± 0.22	390
2-Methylbutyraldehyde	/	659	Fruity, Nuts, Coffee, Caramel	0.001	0.58 ± 0.02	582.98	0.22 ± 0.04	217.08	1.04 ± 0.13	1044.82	1.38 ± 0.42	1376.81	0.27 ± 0.06	270.24	0.45 ± 0.09	446.4	0.55 ± 0.12	553.57
Benzaldehyde	959	961	Almond, Fruity	0.085	0.43 ± 0.02	5.09	0.24 ± 0.02	2.88	0.36 ± 0.03	4.22	0.5 ± 0.16	5.9	1.32 ± 0.27	15.51	0.37 ± 0.05	4.34	0.49 ± 0.12	5.79
Phenylacetaldehyde	1042	1043	Rose-like, Sweet	0.00072	0.7 ± 0	968.47	0.73 ± 0.02	1007.01	1.06 ± 0.08	1465.66	1 ± 0.28	1383.37	0.69 ± 0.25	965.12	0.7 ± 0.14	975.01	1.59 ± 0.23	2209.71
Isobutyric acid	768	785	Burnt, Butter, Cheese	0.0054	0.13 ± 0.02	24.75	0.15 ± 0.03	28.57	0.19 ± 0.04	34.35	0.16 ± 0.07	29.25	ND	ND	0.02 ± 0.01	3.64	0.1 ± 0.03	17.97
Isovaleric acid	863	867	Cheese, Pungent	0.0018	0.46 ± 0.05	258.02	0.56 ± 0.04	312.18	0.9 ± 0.13	500.67	1.97 ± 0.13	1095.58	1.33 ± 0.17	736.69	1.17 ± 0.17	648.63	1.25 ± 0.32	692.5
2-Methyl butyric acid	871	873	Butter, Cheese, Fermented, Sour	0.02	0.25 ± 0.06	12.5	0.24 ± 0.05	12	0.19 ± 0.04	9.73	0.34 ± 0.03	17.11	0.13 ± 0.04	6.29	0.03 ± 0.01	1.62	0.2 ± 0.1	9.78
Ethyl Hexanoate	996	998	Brandy, Fruit Gum, Pineapple	0.003	0.05 ± 0.01	15.46	ND	ND	ND	ND	0.09 ± 0.02	30.78	ND	ND	1.24 ± 0.22	413.85	0.04 ± 0.01	13.51
Ethyl benzoate	1170	1171	Chamomile, Celery, Fat, Flowers, Fruit	0.0006	0.45 ± 0.02	746.04	0.18 ± 0.03	304.68	ND	ND	0.75 ± 0.08	1249.65	0.31 ± 0.04	515.51	0.81 ± 0.15	1349.16	0.38 ± 0.08	640.72
Ethyl phenylacetate	1241	1244	Floral, Fruit, Honey, Rose	0.0033	0.53 ± 0.02	160.45	0.82 ± 0.16	249.66	ND	ND	0.67 ± 0.05	202.65	0.36 ± 0.05	109.25	1.12 ± 0.23	339.3	0.58 ± 0.07	176.61
Diethyl Phthalate	1587	1585	NF	0.33	3.61 ± 0.03	10.95	1.33 ± 0.24	4.04	1.24 ± 0.08	3.75	1.37 ± 0.11	4.16	1.49 ± 0.16	4.52	1.53 ± 0.4	4.64	0.8 ± 0.46	2.42
Ethyl Acetate	/	612	Fruity, Grape, Cherry, Aromatic	0.88	11.37 ± 0.02	12.92	5.47 ± 0.56	6.21	7.31 ± 0.18	8.31	15.8 ± 0.86	17.95	15.48 ± 0.97	17.59	11.89 ± 1.72	13.51	10.84 ± 1.2	12.32
Ethyl isobutyrate	750	755	Fruity, Floral	0.00011	0.07 ± 0.01	663.92	0.31 ± 0.04	2774.29	0.08 ± 0.02	727.27	0.26 ± 0.08	2405.92	0.12 ± 0.07	1048.14	0.04 ± 0.02	323.09	0.12 ± 0.08	1097.68
Ethyl isovalerate	849	847	Apple, Fruit, Pineapple, Sour	0.000069	0.04 ± 0	610.54	0.03 ± 0.01	501.77	ND	ND	0.12 ± 0.03	1794.81	0.05 ± 0.02	701.89	0.04 ± 0.03	648.76	0.1 ± 0.04	1498.87
Isoamyl acetate	873	876	Fruity, Floral	0.067	0.07 ± 0.01	0.97	0.22 ± 0.04	3.34	0.06 ± 0.03	0.86	ND	ND	0.92 ± 0.16	13.76	0.02 ± 0.01	0.34	0.25 ± 0.05	3.79
2-Methylbutyl acetate	875	877	Apple, Banana, Pear	0.14	0.04 ± 0	0.28	0.06 ± 0.02	0.45	ND	ND	0.11 ± 0.02	0.82	0.22 ± 0.08	1.57	ND	ND	0.15 ± 0.09	1.05
2-Pentylfuran	988	993	Beans, Fruit	0.019	ND	ND	0.08 ± 0.03	4.08	0.4 ± 0.03	21.09	0.19 ± 0.04	10.02	ND	ND	ND	ND	0.18 ± 0.06	9.51

^a^ Retention index calculated according to an SH-Rxi-5Sil MS column. ^b^ Retention index reported in the reference standards (https://webbook.nist.gov/chemistry/, accessed on 23 December 2024). ^c^ Odor: Odor descriptions were obtained from literature data (https://www.femaflavor.org/flavor-library, accessed on 23 December 2024). ^d^ Threshold: All the flavor compounds’ thresholds were obtained from relevant literature and a book titled *Compilations of Odor Threshold Values in Air, Water and other Media* [46]. ND: Not detected. NF: Not found.

## Data Availability

The original contributions presented in this study are included in the article/Supplementary Materials; further inquiries can be directed to the corresponding author.

## Supplementary Materials

The following supporting information can be downloaded at https://www.mdpi.com/article/10.3390/foods14050810/s1, Table S1: The contents of the amino acids in the douchi samples; Table S2: The contents of the volatile components between the douchi products.
