# Supplementary material for: Exploring the Core Functional Microbiota Related to Flavor Compounds in Douchi from the Sichuan–Chongqing Region
Source: Foods. 2025 Feb 26;14(5):810. doi: 10.3390/foods14050810 (PMC11898810; doi:10.3390/foods14050810)
Supplement: Supplementary file 1 [file foods-14-00810-s001.zip › foods-3433671-supplementary.pdf]

Supplementary Materials

Table S1. The content of amino acids in douchi samples (g/100g).

Free Amino Acids	Taste	Threshold (g/100g)	DC-1		DC-2		DC-3		DC-4		DC-5		DC-6		DC-7	
			Content (g/100g)	TAV	Content (g/100g)	TAV	Content (g/100g)	TAV	Content (g/100g)	TAV	Content (g/100g)	TAV	Content (g/100g)	TAV	Content (g/100g)	TAV
Asp	Umami	0.1	2.25 ± 0.22	22.47	2.12 ± 0.34	21.25	2.49 ± 1.12	24.91	2.58 ± 0.67	25.84	2.51 ± 0.87	25.11	1.95 ± 0.61	19.53	2.37 ± 0.78	23.72
Glu	Umami	0.03	3.39 ± 0.28	113.16	3.24 ± 0.28	108.04	4.01 ± 1.87	133.59	4.20 ± 1.13	140.16	4.05 ± 1.58	134.99	3.12 ± 0.92	104.12	3.67 ± 1.20	122.33
Thr	Sweet	0.26	0.81 ± 0.17	3.12	0.76 ± 0.11	2.92	0.88 ± 0.42	3.40	0.91 ± 0.28	3.50	0.90 ± 0.31	3.45	0.71 ± 0.25	2.75	0.86 ± 0.27	3.30
Ser	Sweet	0.15	1.13 ± 0.11	7.53	0.87 ± 0.08	5.83	1.07 ± 0.51	7.11	1.12 ± 0.31	7.48	1.11 ± 0.45	7.42	0.88 ± 0.25	5.86	1.01 ± 0.34	6.73
Gly	Sweet	0.13	0.88 ± 0.25	6.75	0.80 ± 0.23	6.12	0.95 ± 0.40	7.29	0.95 ± 0.28	7.30	0.97 ± 0.32	7.46	0.71 ± 0.27	5.49	0.86 ± 0.28	6.59
Ala	Sweet	0.06	0.93 ± 0.19	15.57	0.90 ± 0.15	14.96	1.04 ± 0.48	17.28	1.05 ± 0.31	17.46	1.05 ± 0.37	17.58	0.81 ± 0.27	13.49	0.99 ± 0.34	16.49
Val	Bitter	0.04	1.07 ± 0.16	26.71	1.04 ± 0.33	26.04	1.22 ± 0.47	30.40	1.21 ± 0.43	30.30	1.16 ± 0.41	29.12	0.90 ± 0.29	22.50	1.13 ± 0.39	28.22
Ile	Bitter	0.09	0.27 ± 0.05	2.97	0.23 ± 0.04	2.53	0.27 ± 0.14	3.04	0.31 ± 0.08	3.44	0.30 ± 0.10	3.31	0.26 ± 0.09	2.92	0.30 ± 0.15	3.35
Leu	Bitter	0.19	1.03 ± 0.32	5.41	0.95 ± 0.15	4.99	1.12 ± 0.49	5.90	1.12 ± 0.36	5.92	1.10 ± 0.41	5.79	0.80 ± 0.25	4.22	1.05 ± 0.38	5.54
Met	Bitter	0.03	1.93 ± 0.17	64.31	1.66 ± 0.21	55.17	2.05 ± 0.96	68.23	2.10 ± 0.57	69.90	2.01 ± 0.66	66.99	1.44 ± 0.55	47.84	1.91 ± 0.60	63.71
Tyr	Bitter	0.091	0.72 ± 0.16	7.89	0.49 ± 0.08	5.33	0.60 ± 0.38	6.56	0.61 ± 0.12	6.68	0.72 ± 0.28	7.96	0.43 ± 0.17	4.69	0.55 ± 0.18	6.03
Phe	Bitter	0.09	1.09 ± 0.23	12.11	0.98 ± 0.35	10.88	1.20 ± 0.45	13.30	1.26 ± 0.40	14.04	1.13 ± 0.43	12.55	0.89 ± 0.28	9.94	1.10 ± 0.41	12.22
His	Bitter	0.02	0.34 ± 0.15	16.86	0.31 ± 0.07	15.69	0.33 ± 0.15	16.61	0.34 ± 0.09	16.84	0.36 ± 0.12	18.09	0.29 ± 0.10	14.39	0.34 ± 0.12	16.76
Arg	Bitter	0.05	1.17 ± 0.22	23.40	1.05 ± 0.16	21.03	1.08 ± 0.47	21.58	1.35 ± 0.41	27.07	0.82 ± 0.36	16.50	0.97 ± 0.28	19.35	1.22 ± 0.42	24.37
Lys	tasteless	0.05	0.80 ± 0.12	16.03	0.82 ± 0.06	16.47	0.86 ± 0.46	17.13	0.81 ± 0.17	16.16	1.02 ± 0.38	20.47	0.72 ± 0.26	14.36	0.87 ± 0.29	17.33
Cys	tasteless	-	0.08 ± 0.01		0.08 ± 0.02		0.08 ± 0.07		0.10 ± 0.03		0.09 ± 0.04		0.10 ± 0.04		0.09 ± 0.03	
total	-	-	17.89 ± 0.41		16.29 ± 0.52		19.23 ± 0.23		20.03 ± 0.06		19.32 ± 0.33		14.99 ± 0.35		18.31 ± 0.33	

Note: Results were measured in triplicates and shown as Mean ± Standard deviation. Taste threshold value of free amino acid in water.

Table S2. The content of volatile components between the douchi products (g/100g).

Serial Number	Compounds	SI	RI ref [a]	RI cal [b]	the content of volatile compound (mg/kg)						
					DC-1	DC-2	DC-3	DC-4	DC-5	DC-6	DC-7
Alcohol											
A1	Ethanol	98	/	/	11.91 ± 0.02	8.03 ± 0.41	0.56 ± 0.08	9.92 ± 0.84	19.42 ± 1.57	13.33 ± 1.64	6.36 ± 0.91
A2	2-Methyl-1-propanol	97	622	/	0.13 ± 0.01	-	-	-	0.34 ± 0.06	-	0.29 ± 0.07
A3	3-Methyl-1-butanol	96	734	731	0.08 ± 0.01	0.32 ± 0.05	0.04 ± 0.01	0.14 ± 0.04	1.59 ± 0.31	0.04 ± 0.03	0.29 ± 0.1
A4	2-Methyl-1-butanol	92	736	735	0.05 ± 0.01	0.19 ± 0.03	-	0.14 ± 0.01	0.38 ± 0.16	-	0.17 ± 0.1
A5	(R,R)-2,3-Butanediol	90	/	786	0.2 ± 0.02	0.14 ± 0.04	0.11 ± 0.02	0.13 ± 0.06	0.05 ± 0.03	1.77 ± 0.39	1.06 ± 0.14
A6	Linalool	86	1104	1101	0.17 ± 0.01	-	0.25 ± 0.05	-	0.01 ± 0.01	0.68 ± 0.2	0.26 ± 0.09
A7	Phenylethyl Alcohol	98	1114	1114	0.07 ± 0.01	0.72 ± 0.16	0.06 ± 0.03	0.13 ± 0.03	1.15 ± 0.16	0.09 ± 0.04	0.15 ± 0.06
A8	3-Methyl-2-Heptanol	83	/	793	-	0.1 ± 0.02	-	-	-	-	-
A9	1-Octen-3-ol	85	986	982	-	1.13 ± 0.08	-	0.31 ± 0.04	0.85 ± 0.11	-	0.27 ± 0.08
A10	Benzyl alcohol	92	1034	1038	-	0.06 ± 0.01	-	-	-	-	-
A11	2-Furanmethanol	97	852	855	-	-	0.17 ± 0.05	0.08 ± 0.04	-	-	-
A12	3-Octanol	94	995	998	-	-	-	-	0.14 ± 0.07	-	-
Phenols											
B1	Maltol	96	1088	1107	0.15 ± 0.02	0.61 ± 0.06	0.44 ± 0.09	0.38 ± 0.12	0.12 ± 0.1	0.4 ± 0.08	0.21 ± 0.14
B2	Butylated Hydroxytoluene	93		1503	0.04 ± 0.01	-	-	-	-	-	-
B3	Guaiacol	89	1096	1086	-	0.09 ± 0.02	0.15 ± 0.03	0.2 ± 0.11	0.18 ± 0.08	-	0.08 ± 0.04
B4	4-Ethylphenol	90	1505	1178	-	-	-	-	0.11 ± 0.03	-	-
B5	4-Ethyl-2-methoxyphenol	92	1282	1275	-	-	-	-	0.04 ± 0.02	-	-
Aldehydes											
C1	Isobutyraldehyde	96	552	/	0.18 ± 0.01	-	0.24 ± 0.07	0.34 ± 0.03	0.11 ± 0.03	0.16 ± 0.05	0.18 ± 0.07
C2	Isovaleraldehyde	96	649	/	0.27 ± 0.02	0.32 ± 0.02	0.68 ± 0.12	0.64 ± 0.06	0.21 ± 0.07	0.35 ± 0.07	0.78 ± 0.22
C3	2-Methylbutyraldehyde	95	659	/	0.58 ± 0.02	0.22 ± 0.04	1.04 ± 0.13	1.38 ± 0.42	0.27 ± 0.06	0.45 ± 0.09	0.55 ± 0.12
C4	Furfural	94	830	828	0.08 ± 0.01	-	-	0.13 ± 0.08	-	0.07 ± 0.04	0.35 ± 0.29
C5	Benzaldehyde	96	961	959	0.43 ± 0.02	0.24 ± 0.02	0.36 ± 0.03	0.5 ± 0.16	1.32 ± 0.27	0.37 ± 0.05	0.49 ± 0.12
C6	Phenylacetaldehyde	96	1043	1042	0.7 ± 0	0.73 ± 0.02	1.06 ± 0.08	1 ± 0.28	0.69 ± 0.25	0.7 ± 0.14	1.59 ± 0.23
C7	2-Phenyl-2-Butenal	92	1268	1268	0.05 ± 0	0.05 ± 0.03	-	-	0.11 ± 0.04	0.05 ± 0.06	0.08 ± 0.08
C8	4-Methyl-2-Phenyl-2-Pentenal	85	/	1366	0.02 ± 0.01	-	0.02 ± 0.01	0.04 ± 0.03	-	-	0.01 ± 0.01
C9	Cocal	97	1486	1485	0.08 ± 0.01	0.07 ± 0.03	0.1 ± 0.02	0.16 ± 0.06	-	0.03 ± 0.02	0.08 ± 0.03
C10	Acetaldehyde	94	/	/	-	0.22 ± 0.04	0.2 ± 0.03	0.21 ± 0.03	0.26 ± 0.06	0.22 ± 0.04	0.26 ± 0.06
C11	1-Nonanal	88	1102	1102	-	0.06 ± 0.04	-	-	0.04 ± 0.02	-	0.07 ± 0.05
C12	3-Methyl-2-butenal	89	782	780	-	-	-	0.02 ± 0.01	-	-	-
C13	2-Isopropyl-5-methylhex-2-enal	94	1106	1101	-	-	-	0.11 ± 0.03	-	-	-
Acids											
D1	Isobutyric acid	94	758	768	0.13 ± 0.02	0.15 ± 0.03	0.19 ± 0.04	0.16 ± 0.07	-	0.02 ± 0.01	0.1 ± 0.03

D2	Butyric Acid	87	790	802	0.12 ± 0.01	-	0.08 ± 0.02	-	-	-	-
D3	Isovaleric acid	94	867	863	0.46 ± 0.05	0.56 ± 0.04	0.9 ± 0.13	1.97 ± 0.13	1.33 ± 0.17	1.17 ± 0.17	1.25 ± 0.32
D4	Acetic acid	98	645	/	-	2.1 ± 0.2	3.64 ± 0.53	-	-	-	-
D5	2-Methyl butyric acid	87	872	871	0.25 ± 0.06	0.24 ± 0.05	0.19 ± 0.04	0.34 ± 0.03	0.13 ± 0.04	0.03 ± 0.01	0.2 ± 0.1
D6	Formic acid	88	/	/	-	-	0.12 ± 0.02	0.23 ± 0.09	-	-	-
D7	4-Methylvaleric acid	87	949	967	0.1 ± 0.01	-	-	-	-	-	-
Ketone											
E1	Acetone	96	503	/	1.47 ± 0.02	-	1.49 ± 0.06	1.41 ± 0.23	-	0.42 ± 0.07	0.64 ± 0.1
E2	Acetophenone	93	1069	1064	-	0.15 ± 0.03	0.08 ± 0.02	0.08 ± 0.05	-	-	-
E3	2-Heptanone	94	889	886	-	-	0.06 ± 0.03	-	-	-	-
E4	2,3-Octanedione	85	986	1024	-	-	-	0.08 ± 0.02	-	0.05 ± 0.02	0.07 ± 0.02
E5	2,3-Butanedione	92	593	/	-	-	-	-	-	0.05 ± 0.01	-
E6	2-Hydroxy-3-pentanone	85	/	706	-	-	-	-	-	0.08 ± 0.03	-
E7	L-Fenchone	84	1087	1086	-	-	-	-	-	0.05 ± 0.02	-
Esters											
F1	Methyl acetate	96	522	/	0.29 ± 0.02	-	0.34 ± 0.06	0.58 ± 0.22	0.53 ± 0.05	0.37 ± 0.05	0.44 ± 0.07
F2	Ethyl Acetate	97	612	/	11.37 ± 0.02	5.47 ± 0.56	7.31 ± 0.18	15.8 ± 0.86	15.48 ± 0.97	11.89 ± 1.72	10.84 ± 1.2
F3	Ethyl propionate	94	706	706	0.1 ± 0.02	-	-	0.22 ± 0.09	0.08 ± 0.02	-	-
F4	Ethyl isobutyrate	97	755	750	0.07 ± 0.01	0.31 ± 0.04	0.08 ± 0.02	0.26 ± 0.08	0.12 ± 0.07	0.04 ± 0.02	0.12 ± 0.08
F5	Ethyl butyrate	93	802	798	0.19 ± 0	-	-	-	-	-	-
F6	Ethyl 2-methylbutyrate	96	842	845	0.05 ± 0	0.15 ± 0.06	-	0.27 ± 0.08	0.19 ± 0.03	0.02 ± 0.01	0.21 ± 0.05
F7	Ethyl isovalerate	95	859	849	0.04 ± 0	0.03 ± 0.01	-	0.12 ± 0.03	0.05 ± 0.02	0.04 ± 0.03	0.1 ± 0.04
F8	Isoamyl acetate	93	876	873	0.07 ± 0.01	0.22 ± 0.04	0.06 ± 0.03	-	0.92 ± 0.16	0.02 ± 0.01	0.25 ± 0.05
F9	2-Methylbutyl acetate	91	880	875	0.04 ± 0	0.06 ± 0.02	-	0.11 ± 0.02	0.22 ± 0.08	-	0.15 ± 0.09
F10	Ethyl Hexanoate	93	996	996	0.05 ± 0.01	-	-	0.09 ± 0.02	-	1.24 ± 0.22	0.04 ± 0.01
F11	Ethyl benzoate	96	1170	1170	0.45 ± 0.02	0.18 ± 0.03	-	0.75 ± 0.08	0.31 ± 0.04	0.81 ± 0.15	0.38 ± 0.08
F12	Ethyl phenylacetate	96	1243	1241	0.53 ± 0.02	0.82 ± 0.16	-	0.67 ± 0.05	0.36 ± 0.05	1.12 ± 0.23	0.58 ± 0.07
F13	Linalyl acetate	96	1251	1248	0.11 ± 0.01	-	0.04 ± 0.02	-	-	1.41 ± 0.42	0.04 ± 0.02
F14	Diethyl Phthalate	96	1585	1587	3.61 ± 0.03	1.33 ± 0.24	1.24 ± 0.08	1.37 ± 0.11	1.49 ± 0.16	1.53 ± 0.4	0.8 ± 0.46
F15	Ethyl caprylate	92	1196	1193	-	0.04 ± 0.01	-	0.1 ± 0.02	-	0.05 ± 0.02	0.08 ± 0.04
F16	Phenethyl acetate	88	1256	1253	-	0.06 ± 0.02	-	-	0.13 ± 0.04	-	-
F17	Isobutyl acetate	94	763	767	-	-	-	0.17 ± 0.07	0.22 ± 0.04	-	0.37 ± 0.15
F18	Ethyl lactate	98	815	810	-	-	-	-	0.44 ± 0.13	0.6 ± 0.11	-
F19	Ethyl 3-methylthiopropionate	95	1098	1097	-	-	-	-	0.14 ± 0.04	0.03 ± 0.01	0.02 ± 0.01
F20	Methyl hexanoate	91	924	921	-	-	-	-	-	0.03 ± 0.02	-
F21	Methyl phenylacetate	88	1179	1174	-	-	-	-	-	0.05 ± 0.01	-
F22	Diethyl succinate	89	1181	1176	-	-	-	-	-	0.04 ± 0.02	-
Pyrazines											
G1	2-Methylpyrazine	94	826	820	0.12 ± 0.02	0.09 ± 0.04	0.3 ± 0.04	0.22 ± 0.04	-	0.04 ± 0.02	0.11 ± 0.03

G2	2,6-Dimethylpyrazine	95	916	909	0.48 ± 0.02	0.31 ± 0.04	-	0.78 ± 0.04	-	0.12 ± 0.04	0.35 ± 0.16
G3	2,5-Dimethyl pyrazine	99	913	910	-	-	0.87 ± 0.06	-	0.12 ± 0.03	-	0.06 ± 0.04
G4	2-Ethyl-6-methylpyrazine	88	997	995	-	-	0.06 ± 0.02	0.05 ± 0.04	-	-	0.12 ± 0.04
G5	Trimethyl-pyrazine	91	1005	999	-	-	0.14 ± 0.04	0.1 ± 0.04	-	-	-
G6	Pyrazine	97	734	726	-	-	0.06 ± 0.03	-	-	-	-
G7	3-Ethyl-2,5-diMethylpyrazine	90	1078	1074	-	-	-	-	-	-	0.04 ± 0.02
G8	Tetramethylpyrazine	97	1087	1083	-	-	-	-	-	-	0.03 ± 0.01
Others											
H1	2,5-Dimethylfuran	92	706	703	0.07 ± 0.01	-	0.12 ± 0.02	0.07 ± 0.03	-	0.07 ± 0.02	0.08 ± 0.04
H2	2-Methylbutyronitrile	90	717	716	0.05 ± 0.01	-	-	-	-	-	-
H3	3-Methylbutanenitrile	94	730	723	0.11 ± 0.01	-	0.03 ± 0.03	-	-	-	-
H4	D-Limonene	91	1028	1027	0.03 ± 0.01	-	0.04 ± 0.01	-	-	0.5 ± 0.12	0.08 ± 0.03
H5	2-Acetyl pyrrole	93	1063	1067	0.28 ± 0.02	0.21 ± 0.03	0.59 ± 0.06	0.82 ± 0.13	-	0.29 ± 0.1	0.28 ± 0.11
H6	Dimethoxybenzene	87	1175	1166	0.05 ± 0.01	-	-	-	-	-	-
H7	Ethenylsulfanylethene	85	/	1220	0.04 ± 0.01	-	-	-	-	-	-
H8	4-Allylanisole	90	1208	1287	0.28 ± 0.01	0.04 ± 0.01	-	-	-	3.04 ± 0.33	0.13 ± 0.07
H9	2-Pentylfuran	89	987	988	-	0.08 ± 0.03	0.4 ± 0.03	0.19 ± 0.04	-	-	0.18 ± 0.06
H10	2-Ethylfuran	89	702	/	-	-	0.03 ± 0.01	-	-	-	-
H11	Dimethyl Disulfide	96	742	736	-	-	0.2 ± 0.03	-	-	-	-
H12	Toluene	95	761	759	-	-	0.07 ± 0.02	0.04 ± 0.02	-	-	-
H13	Dimethyl trisulfide	93	956	965	-	-	0.23 ± 0.1	0.15 ± 0.07	-	-	-
H14	Trans-Anethole	93	1284	1287	-	-	0.12 ± 0.05	-	-	-	-
H15	2-Acetylfuran	85	914	907	-	-	-	0.08 ± 0.04	-	-	-
H16	Myrcene	95	991	987	-	-	-	-	-	0.17 ± 0.04	-
H17	Alpha-Terpinene	94	1015	1015	-	-	-	-	-	0.06 ± 0.04	-
H18	trans-.beta.-Ocimene	95	1045	1035	-	-	-	-	-	0.05 ± 0.02	-
H19	.gamma.-Terpinene	95	1060	1057	-	-	-	-	-	0.14 ± 0.04	-
H20	Terpinolene	87	1089	1084	-	-	-	-	-	0.03 ± 0.01	-
H21	β-Caryophyllene	85	1427	1425	-	-	-	-	-	0.01 ± 0.01	-
H22	2-Butoxyethanol	85	/	707	-	-	-	-	-	-	0.11 ± 0.04
H23	Ethylbenzene	96	857	856	-	-	-	-	-	-	0.17 ± 0.05
H24	m-Xylene	93	862	866	-	-	-	-	-	-	0.09 ± 0.05
H25	Styrene	98	890	889	-	-	-	-	-	-	1.39 ± 0.58
H26	3-Phenylfuran	87	1224	1224	-	-	-	-	-	-	0.04 ± 0.03

[a] Retention index calculated according to an SH-Rxi-5Sil MS column. [b] Retention index reported in the reference standards (https://webbook.nist.gov/chemistry/, accessed on 23 December 2024).
